# Comparing the efficacy of targeted spinal cord stimulation (SCS) of the dorsal root ganglion with conventional medical management (CMM) in patients with chronic post-surgical inguinal pain: the SMASHING trial

**DOI:** 10.1186/s12893-018-0349-8

**Published:** 2018-03-27

**Authors:** Frederique M. U. Mol, Rudi M. Roumen, Marc R. Scheltinga

**Affiliations:** Department of Surgery, Maxima Medical Center, de Run 4600, PO Box 7777, 5500 MB Veldhoven, The Netherlands

**Keywords:** Post-surgical inguinal pain (PSIP), Spinal cord stimulation (SCS), Dorsal root ganglion (DRG)

## Abstract

**Background:**

A significant number of patients who undergo a standard inguinal hernia repair or a Pfannenstiel incision develop chronic (> 3 months) post-surgical inguinal pain (PSIP) due to nerve entrapment. If medication or peripheral nerve blocks fail, surgery including neurectomies may offer relief. However, some patients do not respond to any of the currently available remedial treatment modalities.

Targeted spinal cord stimulation (SCS) of the dorsal root ganglion (DRG) is a relatively new type of therapy that has a potential to significantly reduce chronic PSIP. The Axium® SCS System (Spinal Modulation Inc., NY, USA) has been shown to be safe and successful in small cohorts of PSIP patients.

Aim of this study is to evaluate targeted spinal cord stimulation therapy in patients with PSIP.

**Methods:**

A prospective, multicentre, randomized controlled trial with optional one-way crossover will assess the efficacy of the Axium® SCS system for the treatment of PSIP. Seventy-eight patients with intractable PSIP following open hernia repair or Pfannenstiel incision who did not respond favorably to previous pain treatment regimens including a neurectomy will be randomized to either an Axium® SCS arm or a control arm receiving only conventional medical management (CMM).

Primary outcome is the difference in percentage of subjects with ≥50% pain relief after 6 months using a Numerical Pain Rating Scale (NPRS). Data are collected using a daily pain/sleep diary and a number needed to treat (NNT) analysis is performed. Various secondary outcomes will be collected.

**Discussion:**

Targeted SCS stimulation of the DRG using the Axium® SCS system will possibly offer significant pain reduction in patients with PSIP who are refractory to other treatment modalities.

**Trial registration:**

The study protocol is registered at the NIH Clinical Trials Registry (http://clinicaltrials.gov, ClinicalTrials.gov identifier: NCT02349659) on January 29, 2015.

## Background

Herniorrhaphies are performed 800.000 times a year in the U.S. A male person in the industrialized world faces an up to 27% lifetime risk of requiring surgery for his inguinal hernia [1]. Some 10–12% of these patients were found to report moderate to severe chronic pain after the operation [2–4]. Chronic postherniorrhapy inguinal pain may be caused by nociceptive, inflammatory or neuropathic changes, the latter depending on whether inguinal nerves are affected. A comparably related pain syndrome may occur after nerve entrapment following a lower abdominal pfannenstiel incision [5]. Both pain entities are also referred to as chronic post-surgical inguinal pain (PSIP) syndromes.

Recently, a consensus was proposed concerning the management of chronic postherniorrhapy inguinal pain [6]. If non-surgical options failed, a triple neurectomy with inguinal nerves removal via an inguinal approach is suggested. Success rates range from 50 to 100%, depending on surgical technique (open or laparoscopic) and definitions of outcome measures [7–9]. However, the suggested consensus did not address alternative treatment options once a patient was an all options non-responder. Interestingly, a recent study showed that the number needed to harm, defined as patients who had an increase > 25% of pain compared to preoperatively, was 1 of 8 patients at a 3 year follow up when a selective neurectomy accompanied by mesh removal was performed. [10]

Currently, few therapeutic options are available for patients demonstrating limited (or no) success after neurectomy. A promising new treatment tool is spinal cord stimulation (SCS) of the dorsal root ganglion. This novel variant of traditional SCS is claimed to effectively target the groin and has proved to be a safe and efficaous therapy in small, heterogeneous case series of patients with PSIP [11, 12]. In order to evaluate whether this therapy is an effective treatment option for PSIP, a randomized clinical trial comparing SCS to conventional medical management is proposed. [13] Aim of the present article is to report on characteristics regarding the set-up of this randomized clinical trial.

## Methods

### Study design

The SMASHING (Spinal Modulation After failed Surgery for chronic pain following Hernia treatment in INGuinal area) trial is a randomized, cross-over, multicenter study. The protocol is approved by the Regional Ethics Committee of the Máxima Medical Center, Veldhoven, The Netherlands (no. 1435). The objective is to evaluate the efficacy of SCS of the DRG with an Axium® SCS System of Spinal Modulation Inc. in patients with post-surgical pain after a hernia repair or pfannenstiel incision who did not favorably respond to a neurectomy. Clinical results of the Axium arm are compared with those in a CMM arm. Patient enrolment has started in February, 2015. The study protocol is registered at the NIH Clinical Trials Registry (http://clinicaltrial.gov, ClinicalTrials.gov identifier: NCT02349659).

### Inclusion criteria

Adults (18 years or older) of either gender suffering from persistent inguinal pain (minimum duration of 6 months) following either open or laparoscopic inguinal hernia repair surgery or a Pfannenstiel incision who have subsequently undergone and failed to respond to a neurectomy procedure will be considered for inclusion. Failure to benefit from additional treatment options such as peripheral nerve blocks, physical therapy, minimally invasive interventional pain procedures and medical regimens with at least a tricyclic antidepressant or similar neuropathic pain agents is required. Patients should have a minimum daily average baseline pain rating of 5 out of 10 in the inguinal area on an 11-point NPRS. Patients should be able to operate the device. Inclusion criteria as set out by the Dutch Neuromodulation Society should be met. A psychologist will screen patients and assess for suitability within a multidisciplinary team including an anesthesiologist or pain specialist and a surgeon-herniologist. [6]

Patients are excluded if there are contra-indications for device implantation such as pregnancy, ongoing infection, coagulation disorders, previous spinal surgery at or between vertebral levels T10-L2, the presence of other active implantable devices or a condition which will require MRI investigations. Patients are not allowed to undergo injection therapy or radiofrequency treatment at the target neural structure in the 3 months prior to inclusion. An escalating or changing pain condition and a diagnosis of cancer in the past 2 years are also considered as exclusion criteria.

### Randomization

Patients are considered for the trial if all criteria are satisfied. After suitability within is confirmed by the multidisciplinary team, informed consent is signed after patient is adequately informed and has agreed upon participation. Patients will be randomized in a 1:1 ratio to either arm. An independent statistician will generate and hold the allocation sequence, concealed from those involved in assessing eligibility and recruiting subjects.

### Interventions

**Fig. 1:**
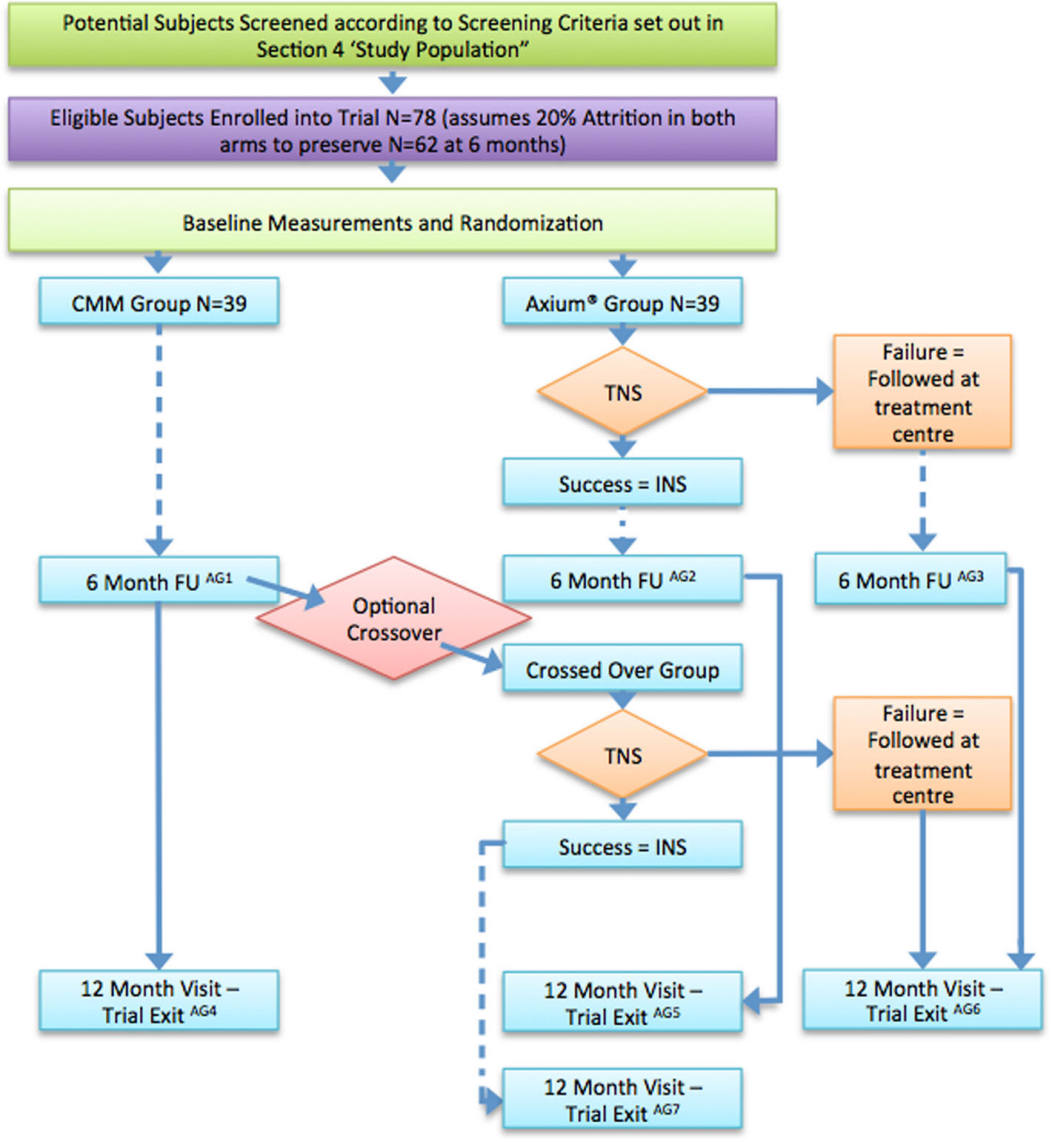
Basic design and follow-up schedule. Seven analysis groups are indicated (‘AG’) to refer to groups at several time points

The SCS group (Axium® Group, Fig. 1) receives an Axium® SCS System within 4 weeks after randomization. Implantation is performed in one of the participating centers by an experienced anesthesiologist or neurosurgeon. Electrodes are placed in the epidural space near the targeted DRG under sedation in a surgical theatre, using a transforaminal, fluoroscopy checked approach. During the procedure, the patient is asked if paresthesia elicited by stimulation covers the painful area. If so, electrodes are connected to an external stimulator if a trial phase is deemed needed. After a successful trial (defined as reaching adequate pain relief upon stimulation), a fully implantable device will be placed in a second session some 2–3 weeks later on as earlier suggested. [11, 12] After implantation, patient will be subjected to a standard follow-up schedule as dictated by the implanting center plus additional trial visits if needed.

After randomization, the CMM (CMM group, Fig. 1) will continue with prior pain treatments as previously initiated by their general practitioner or pain specialist. This regimen will generally comprise physical therapy and medications such as a tricyclic antidepressant (amitriptyline) and/or anticonvulsants (pregabalin, gabapentin, valproic acid), opioids or other analgesics, as is the standard protocol for neuropathic pain. Use and adjustment of pain medication is allowed and monitored. There are no restrictions to dosage and sort of pain medication including opioids.

However, interventional pain techniques are not allowed during this time period. If (pulsed) radiofrequency, epidural or peripheral injection or infusion therapy with analgesic agents or any other form of neurostimulation (traditional SCS, Deep Brain Stimulation, TENS) are performed, these treatments are considered as a protocol violation. After 6 months, patients will be offered to cross-over to the Axium ® Group. They will then receive treatment and follow-up as previously described.

### Collection of data and clinical follow-up

Patients of both groups will undergo a follow-up visit after 2 weeks, 1 month, 3 months, 6 months and 12 months postrandomisation. Standardized questionnaires investigating quality of life, physical impairment, health resources utilization, subject satisfaction and adverse events will be completed. Validated questionnaires such as the EQ-5D, BPI, NPSI (Neuropathic Pain Symptoms Inventory) and a 7 point Likert Scale will be used. Pain and sleep will be evaluated using a pain-sleep interference diary containing an NPRS and DSIS over a 7-day period. Measures of average, worst and least pain during the previous 24 h are obtained using this diary. Primary analysis is conducted on average pain intensity scores. Physical examination will be performed in order to assess changes in neuropathic pain symptoms.

### Study endpoints

The primary outcome is percentage of subjects experiencing a minimal 50% reduction in average daily pain intensity as measured using the average NPRS as noted in pain diaries in the Axium® group as compared to the CMM group. Group differences in the change in pain intensity scores (NPRS) at the 6-month FU visit are also calculated. The analysis will be done following an Intention to Treat (ITT) principle.

**Fig. 2:**
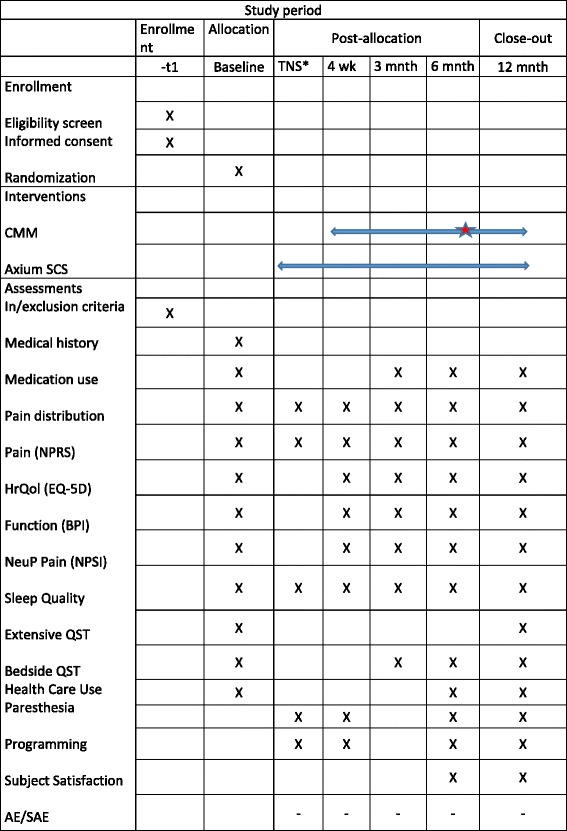
SPIRIT Flow chart. The red star marks the cross-over option for the CMM group. *TNS: Trial stimulation phase (1 week)

Secondary outcomes are an ‘as treated’ analysis of the percentage of subjects experiencing a minimal 50% reduction in average daily pain intensity (NPRS) at 6 months and the mean difference in the change in pain intensity scores (NPRS) between the successfully trialed subjects with the Axium® arm as opposed to the CMM arm. This ‘as treated’ analysis is important as it reflects clinical convention i.e. utilization of a therapy trial (TNS) to improve changes of a successful outcome with the fully implantable system. A number needed to treat (NNT) analysis at the 6-months time point for ≥30% and ≥50% pain relief is also performed. However, reduction in pain intensity is not the only clinically relevant outcome in chronic pain patients. Changes in disability, pain interference, neuropathic pain symptoms, sleeping disorders and health resources utilization are also investigated using validated questionnaires at baseline and each follow-up visit. (Fig. 2).

Total study duration is anticipated to be 2 years.

### Sample size

Sample size estimation is based on a ‘responder’ analysis as recommended by the Initiative on Methods, Measurement, and Pain Assessment in Clinical Trials (IMMPACT) group recommendations [14]. The estimated proportion of responders (≥ 50% reduction in pain NPRS) in the CMM group is 10%, based upon data generated by a RCT on SCS in failed back surgery syndrome (FBSS) [15]. Expected effect size in the Axium® group is extrapolated from published literature [11, 12]. The targeted effect size is a difference in proportion responding to treatment (Axium® vs. CMM) at the 6-month time point of 40%; i.e., 50% responding in Axium® group vs. 10% in the CMM group. With 90% power and 2p = 0.05, using a normal approximation with continuity correction, 31 subjects are required in each group at the 6 months primary endpoint analysis follow up point. With an allowance for attrition of 20% in both arms at 6-month follow-up, a total of 78 subjects is recruited (62/0.8 = 78). Sample size estimation was conducted using Stata software (version 13.1; StataCorp, College Station, TX). Figures were verified using Medcalc® software (v.14.8). There are no specific data to judge the effect size of CMM or the drop out rates in this specific study population.

### Statistical analysis

The primary analysis is a comparison of the proportion of responders (≥ 50% pain relief) in the Axium® versus CMM group at the 6-months time point. The mean difference between proportions and 95% confidence intervals are calculated. A risk difference is obtained using generalized linear modeling with a binomial distribution and an identity link. Difference between groups in the change in pain score (6-months minus baseline), adjusted for any chance imbalance between groups at baseline (ANCOVA model) are also calculated. The mean treatment effect (Axium® minus CMM) together with its 95% confidence interval is obtained. NNT will also be calculated as the reciprocal of the difference in proportions of responders (Axium® minus CMM) and presented with its 95% confidence interval. All analyses are conducted both ‘as randomized’ (intention to treat principle) and ‘as treated’ (secondary).

## Discussion

The results of the presently proposed ´SMASHING´ trial may shed light on a possible viable alternative treatment option once other treatments have failed in therapy resistant patients with PSIP. A very recent consensus protocol does not address this group [6]. Based on a retrospective analysis of 10 CPIP patients who were implanted with DRG stimulation leads, the technique is very promising. Eight of these 10 patients reported > 50% pain relief whereas a 77% mean VAS reduction was attained. [11] However, it is obvious that a proper RCT is required using a cross-over design. Firstly, a placebo controlled setting is inappropriate as it is clearly noticeable for patients if a sham device is implanted because of the absence of paresthesia. Secondly, ethics direct that the control group is not to be denied access to the therapy for which a cross-over is offered. Thirdly, a control group is heterogeneous because of the often individually tailored treatments for these therapy-resistant patients. This heterogeneity however mirrors daily clinical practice, and therefore generalization of the forthcoming results will be realistic. Moreover, possible confounding variables are controlled with randomization. The PROCESS study used a similar construction [15].

It must be appreciated that the validation of various outcomes is of utmost importance in pain treatment studies. Pain reduction scores are subjective whereas the clinical relevance of a 30% or 50% pain reduction is debatable. The presently proposed outcome measurements are linked to improved patient satisfaction, diminished medication usage, daily functioning and sleeping quality and a positive expert’s opinion regarding patient’s improvement [16–19]. We will address these components as well as other secondary outcomes as all of these issues are indispensable for a thorough efficacy analysis of any type of novel pain treatment.

### Update

Per 17-Jan-2017, sponsorship of the study has been transferred from St. Jude / Abbott to the Maxima Medical Center and primary investigator due to a slow inclusion rate. Analysis of the preliminary results of 18 patients enrolled from March 2015 to November 2016 is currently performed using repeated measures ANOVA on the primary outcome (number of patients with > 50% pain reduction and individual pain reduction). The original protocol did not describe regulations for interim analysis, but the authors will adhere to Bayesian approach as described in Li and Gates, 2013 [20].

## Abbreviations

BPI: Brief Pain Inventory
CMM: Conventional Medical Management
CPIP: Chronic Post-herniorrhaphy Inguinal Pain
DRG: Dorsal Root Ganglion
DSIS: Daily Sleep Inventory Score
ITT: Intention To Treat
NNT: Number Needed to Treat
NPRS: Numerical Pain Rating Score
PSIP: Post Surgical Inguinal Pain
SCS: Spinal Cord Stimulation
TENS: Transcutaneous Electrical Nerve Stimulation
TNS: Temporary Neuro Stimulation
